# Associations of Nausea and Vomiting of Pregnancy with Maternal and Fetal Outcomes

**DOI:** 10.3390/jcm14124300

**Published:** 2025-06-17

**Authors:** Maria De Bonis, Giulia Cimino, Ilaria Ianes, Eugenia Costantini, Claudia d’Abate, Gabriele Centini, Lucia Lazzeri, Errico Zupi, Francesco Giuseppe Martire

**Affiliations:** Department of Molecular and Developmental Medicine, Obstetrics and Gynecological Clinic, University of Siena, 53100 Siena, Italy; mariadebonis@gmail.com (M.D.B.); giulia.cimino@student.unisi.it (G.C.); ilaria.ianes@gmail.com (I.I.); eugenia.costantini22@gmail.com (E.C.); claudiadabate94@gmail.com (C.d.); centini.gabriele@gmail.com (G.C.); lucialazzeri79@gmail.com (L.L.); francescogmartire@libero.it (F.G.M.)

**Keywords:** hyperemesis gravidarum, nausea and vomiting, pregnancy, quality of life

## Abstract

**Background:** Nausea and Vomiting in Pregnancy (NVP) and Hyperemesis Gravidarum (HG) are common pregnancy-related conditions that can significantly impair maternal quality of life and, in severe cases, impact pregnancy outcomes. This study aimed to assess the prevalence of NVP and HG, evaluate their association with pregnancy progression and neonatal outcomes, and investigate the role of pharmacological therapy. **Methods:** A prospective observational study was conducted at the University Hospital of Siena between September 2023 and September 2024. Seventy-nine pregnant women aged 28–34 years were enrolled and followed throughout pregnancy. Symptom severity was assessed using the PUQE questionnaire during scheduled outpatient visits. Patients with NVP received pharmacological treatment with doxylamine succinate/pyridoxine hydrochloride. **Results:** Nausea and Vomiting in Pregnancy was reported by 59% of patients, with all cases categorized as mild or moderate; no severe HG cases were observed. Symptoms resolved by the third trimester. A significant association was observed between NVP and a positive family history of the condition (OR: 3.66, 95% CI: 1.20–11.21; *p* = 0.025). Logistic regression analysis also revealed that NVP was associated with an increased risk of gestational hypertension (15% vs. 0%, *p* = 0.04), and a decreased likelihood of gestational diabetes (OR: 0.24, 95% CI: 0.07–0.86) and cesarean section (OR: 0.34, 95% CI: 0.13–0.87). No significant differences were found in neonatal outcomes, including birth weight, Apgar scores, or fetal complications. **Conclusions:** While NVP may influence maternal outcomes, the condition does not significantly affect neonatal health. Early pharmacological treatment improves maternal well-being and may reduce hospitalization rates. Larger multicenter studies are needed to confirm these findings.

## 1. Introduction

Nausea and Vomiting of Pregnancy (NVP) affects up to 80% of pregnant women and is one of the most common causes of hospital admission during pregnancy. Prevalence rates for nausea range from 50% to 80%, with approximately 50% of women experiencing vomiting. NVP is defined as nausea and/or vomiting during pregnancy with onset before 16 weeks of gestation, in the absence of other underlying causes [1].

Hyperemesis Gravidarum (HG) is a severe form of NVP, affecting between 0.3% and 3.6% of pregnant women, and can lead to significant impairment of daily functioning, dehydration, and metabolic disturbances [2]. Although there is no universally accepted definition, the recent literature has proposed criteria to standardize diagnosis. According to Windsor et al., HG is defined by the onset of severe nausea and/or vomiting in early pregnancy (before 16 weeks of gestation), with an inability to eat and/or drink normally, significant disruption of daily activities, and clinical signs of dehydration [3].

In these situations, metabolic disturbances, such as electrolyte imbalance and ketonuria, are common clinical features requiring prompt management [4]. These symptoms often require hospitalization and prompt management with fluid replacement, antiemetics, and nutritional support [5].

The etiology of NVP remains unclear, with these conditions likely being multifactorial. Recent research has identified increased sensitivity to the vomiting hormone, growth differentiation factor-15 (GDF15), as a key mechanism underlying NVP [6]. GDF15 induces loss of appetite, taste aversion, nausea, vomiting, and weight loss. Variations in the GDF15 gene, observed in both familial and sporadic cases, are associated with HG. This is particularly relevant as the activation of placental genes during pregnancy leads to the production of GDF15, with circulating levels peaking in the first half of pregnancy [7].

Risk factors for NVP include young maternal age, female fetus, multiple pregnancy, elevated hCG, gastrointestinal or endocrine disorders, psychiatric comorbidities, and vitamin deficiencies [8].

NVP is associated with hyponatremia, hypokalemia, low serum urea, elevated haematocrit and ketonuria, often resulting in metabolic hypochloremic alkalosis, and in severe cases, metabolic acidosis [9]. NVP can alter thyroid function, liver enzymes, and electrolyte balance, potentially contributing to maternal and fetal complications [10,11].

Therapeutic options for NVP include medications from various pharmacologic classes, and many women require a combination of two or more antiemetics for adequate symptom control [12]. A slow-release combination of doxylamine succinate and pyridoxine hydrochloride—commercially known as Bonjesta^®^ (Europe), Diclegis^®^ (Duchesnay Inc., Princeton, NJ, USA) or Diclectin^®^ (Duchesnay Inc., Blainville, QC, Canada)—is the only treatment specifically approved for NVP in several countries. This combination has been shown to significantly improve the Pregnancy-Unique Quantification of Emesis and Nausea (PUQE) score from baseline to day 15 when compared with a placebo (mean difference −0.90, 95% CI −1.55 to −0.25; *p* = 0.006) [13]. Other antiemetic agents, including antihistamines, dopamine antagonists (such as Metoclopramide), serotonin antagonists (such as ondansetron), and corticosteroids (such as Prednisolone), are also used off-label depending on symptom severity and response to treatment [8].

For hospitalized patients, the primary goal is to stabilize the patient through proper rehydration, electrolyte restoration, and symptom management. Essential interventions include intravenous fluid administration and thiamine supplementation [14].

NVP and HG are known to reduce quality of life, impairing daily functioning and affecting interpersonal relationships. Early diagnosis, effective management, and psychological support are therefore crucial to improving maternal well-being and pregnancy experience, including neonatal bonding [15].

In terms of neonatal outcomes, these conditions have been associated with an increased risk of preterm birth before 37 and 34 weeks, though not below 32 weeks [16]. The literature suggests that severe HG is associated with a higher risk of adverse outcomes compared to milder forms of the condition [17].

The aim of our prospective observational study was to assess the prevalence of NVP and HG within our population, evaluate the impact of these conditions on pregnancy progression and neonatal outcomes, and assess the role of medical therapy.

## 2. Materials and Methods

**Figure 1:**
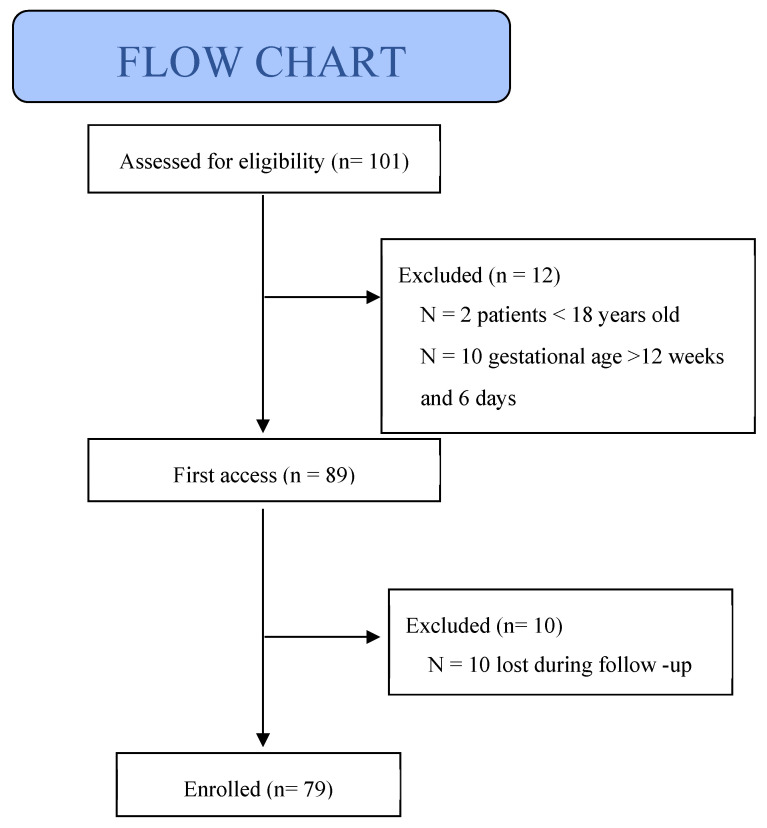
Flow chart of inclusion study population.

This prospective observational study aimed to investigate the potential impact of NVP on maternal and fetal health. Between November 2023 and November 2024, 101 pregnant women were assessed for eligibility at the obstetric outpatient clinics of the University Hospital of Siena; 12 patients did not meet the inclusion criteria and 10 were lost to follow-up. A total of 79 patients were included in the final analysis (Figure 1). Written informed consent was obtained from all participants, and the study was approved by the Local Ethics Committee (protocol no. 24950).

### 2.1. Inclusion and Exclusion Criteria

Women aged >18 years, with either singleton or multiple pregnancies, were eligible for inclusion, regardless of whether conception occurred spontaneously or via assisted reproductive techniques (ART). Pregnant women were excluded if they were <18 years old, had a gestational age >12 weeks and 6 days at their first prenatal visit, had pre-existing conditions that could cause nausea and vomiting (e.g., gastrointestinal or renal disorders), liver diseases, or were unable to understand the study’s purpose due to language barriers. Women unable to commit to regular follow-up visits were also excluded.

In the NVP group, all patients received pharmacological treatment with doxylamine succinate/pyridoxine hydrochloride at a standard initial dosage of 10 mg/10 mg per tablet, with a dose of 2 to 4 tablets taken daily depending on symptom severity and clinical response. The dosage was later adjusted to two administrations per day once symptoms began to regress.

### 2.2. Data Collection

**Figure 2:**
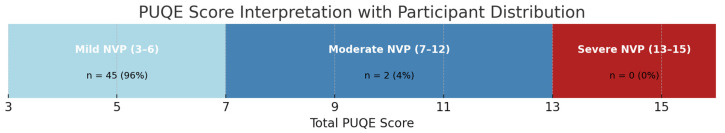
PUQE score interpretation with participant distribution.

Data were collected using the Pregnancy-Unique Quantification of Emesis (PUQE) questionnaire, a validated tool for assessing the severity of NVP. The questionnaire consists of three items: (i) duration of nausea in the past 12 h, (ii) number of vomiting episodes in the past 12 h, and (iii) number of retching episodes in the past 12 h. Each item is scored from 1 to 5 based on severity, with a total score of 4–6 indicating mild NVP, 7–12 indicating moderate NVP, and >13 indicating severe NVP. A Likert-type scale summarizing the PUQE scoring system and our participants’ response distribution has been added to visually support the interpretation of results (Figure 2).

Nausea and Vomiting in Pregnancy (NVP) was defined as the presence of nausea, retching, and/or vomiting occurring during the first trimester of pregnancy and not attributable to other causes. The severity of NVP was assessed using the Pregnancy-Unique Quantification of Emesis and Nausea (PUQE) score, a validated tool that categorizes symptoms as mild (PUQE score 3–6), moderate (7–12), or severe (13–15).

Hyperemesis Gravidarum (HG) was defined as a severe form of NVP characterized by persistent vomiting, weight loss exceeding 5% of pre-pregnancy weight, dehydration, electrolyte imbalance, and/or the need for hospitalization. In this study, the clinical diagnosis of HG was based on a combination of symptom severity (as measured by PUQE), medical records, and clinical judgment.

The PUQE questionnaire was administered by trained medical staff during routine obstetric outpatient visits and completed by patients under medical supervision. All personnel involved in data collection had been previously trained to ensure consistent administration, and the same staff members were involved throughout the entire data collection period to maintain uniformity. Only patients who completed the PUQE questionnaires in their entirety during the visits were included in the final analysis. N = 10 participants who missed follow-up appointments and did not complete the questionnaires were excluded from the study. No patients were excluded due to language barriers, as all participants were able to speak and/or understand Italian and/or English. The collected data were anonymized and entered into a secure database. Data collection occurred at scheduled obstetric visits: in the first trimester (up to 12 + 6 weeks), second trimester (14–20 weeks), early third trimester (28–32 weeks), at 37–38 weeks, and within 7 days postpartum.

### 2.3. Statistical Analysis

The study aimed to evaluate the association between NVP and adverse pregnancy and fetal outcomes. Statistical analyses initially assessed patient characteristics across the entire study population. The 79 pregnant women included were divided into two groups: those with symptoms of NVP and those without. Pre-pregnancy anamnesis, pregnancy complications, and neonatal outcomes were evaluated in both groups.

Clinical and demographic data were analyzed as mean ± standard deviation (SD) for quantitative variables and as percentages and frequencies for qualitative variables. Univariate analysis was performed using the Student’s *t*-test for continuous variables and the Fisher exact test for categorical variables to compare characteristics between groups.

To identify independent risk factors associated with negative outcomes (such as preterm birth, low birth weight, neonatal mortality, etc.), a multivariate logistic regression model was applied, and adjusted for potential confounding factors such as maternal age, parity, and sociodemographic variables. All statistical analyses were performed using IBM SPSS Statistics (latest version available at the time of analysis). Statistical significance was defined as *p* < 0.05. Additionally, 95% confidence intervals were calculated to ensure the robustness of the results. For continuous variables not significantly associated with the outcome, such as maternal age and gestational weight gain, odds ratios were not reported, as categorization was not performed and the interpretation would not be clinically meaningful.

## 3. Results

A total of 79 pregnant women were enrolled, with a mean age of 31 years (range 28–34 years old). The majority of participants were nulliparous, and most pregnancies (94%) were spontaneous. Most cases involved single pregnancies, while 4% were twin pregnancies. Table 1 summarizes the demographic and clinical characteristics of the study population.

Among the 79 patients, 47 (59%) experienced NVP, while 32 (40%) did not. Severity, as assessed using the PUQE questionnaire, showed that during the first trimester, 96% of participants had mild symptoms (score 4–6), 4% had moderate symptoms (score 7–12), and 0% had severe symptoms (score > 13). Accordingly, none of the participants presented symptoms severe enough to meet the clinical criteria for a diagnosis of hyperemesis gravidarum (HG). In the second trimester, the number of affected patients decreased to 15 (19%), all with mild symptoms, and no cases of NVP or HG were observed in the third trimester.

Analysis of maternal characteristics prior to pregnancy revealed no statistically significant differences between the two groups (*p* > 0.05), indicating a homogeneous population. Notably, 13% of participants had a history of thyroid disorders. Pre-pregnancy diabetes and hypertension were rare, each present in only 1% of the population (one individual per condition). These patients were not excluded from the study in order to preserve statistical power given the limited sample size, and to better reflect real-world clinical conditions. Including participants with endocrine comorbidities also allowed us to explore the potential influence of these factors on the presence and severity of NVP within the cohort.

Among the 47 women who experienced NVP, a statistically significant association emerged between NVP and a positive family history (i.e., NVP or HG in first-degree relatives), with an odds ratio of 3.66 (95% CI: 1.20–11.21; *p* = 0.025) suggesting a potential genetic or familial predisposition to the condition. This finding aligns with recent studies suggesting that genetic factors, such as increased susceptibility to the vomiting hormone GDF15, are key predisposing causes for NVP development. Furthermore, in the NVP group, only three patients were from ART pregnancies and all these patients had mild NVP. Particularly, two patients resolved NVP in the first trimester, while one of these patients resolved it in the second trimester.

The study also examined pregnancy-related conditions (Table 2), including gestational hypertension, preeclampsia, eclampsia, cholestasis, gestational hypothyroidism, and gestational diabetes. Logistic regression analysis showed a significantly higher risk of gestational hypertension in women with NVP (15% vs. 0%, *p* = 0.04). Specifically, we also assessed whether persistence of nvp in the second and third trimesters could worsen the prevalence of gestational hypertension, comparing early NVP with continued NVP with no evidence of association (Table 3). Conversely, gestational diabetes and cesarean section were less frequent in the NVP group, with ORs of 0.24 (95% CI: 0.07–0.86; *p* = 0.03) and 0.34 (95% CI: 0.13–0.87; *p* = 0.03), respectively. No other significant differences were found between the NVP and non-NVP groups (*p* > 0.05).

Regarding fetal complications (Table 4), a slightly higher proportion of fetal malformations was observed in the NVP group, although this difference was not statistically significant. No significant differences were found in Apgar scores at 1 and 5 min between the NVP and non-NVP groups (*p* > 0.05). Additionally, NVP did not affect gestational age at birth, fetal sex, or birth weight, with no significant differences between the groups (*p* > 0.05), consistent with the findings on fetal growth outcomes.

Within the NVP group, 49% of patients received treatment during pregnancy, while 10% did not. The use of therapy did not significantly influence the outcomes reported in the study.

## 4. Discussion

NVP is a pregnancy-related condition that typically develops in the first trimester and resolves spontaneously during the second trimester [1]. This condition has a relatively high incidence, and while it affects the quality of life of the affected women, it can also impact their pregnancy, particularly in severe cases, with potential maternal and fetal consequences [18].

This prospective study aimed to evaluate the prevalence of NVP and HG at our center, identify risk factors for the condition, assess the impact of hyperemesis on pregnancy and fetal health, and examine the role of medical therapy.

The estimated incidence of NVP is approximately 70%, and disease severity appears to be inversely related to maternal age [19]. Our findings align with the existing literature, revealing an incidence rate of around 60%. Additionally, in our study population, with a mean age of 31 years, 96% of patients had mild symptoms, with no cases of HG reported.

One potential issue is the need for hospital stay, which increases healthcare costs [11]. Hospitalization is typically required for severe hyperemesis, which can result in plasma electrolyte imbalances [8]. Hyperemesis is the second most common cause of hospitalization during pregnancy [7], but in our study, where the majority of cases were mild, the hospitalization rate was low. Furthermore, early initiation of therapy in our study population may be an effective strategy to reduce inpatient care and healthcare costs [8].

Almost 50% of women with NVP at our center reported a family history of NVP in at least one first-degree relative [20]. Larger population studies are needed to assess the impact of family history compared to patients without NVP. Regarding other common gynecological conditions in reproductive age, such as fibroids, endometriosis, adenomyosis, or polycystic ovary syndrome (PCOS) [21,22], there is no increased risk to present NVP. Current evidence on the relationship between pre-pregnancy gynecological conditions and the development of NVP remains limited.

In agreement with the literature data, our patients undergoing ART also developed NVP, and considering the high maternal age of our group, which should be a protective factor, this confirms that ART itself is a risk factor for developing NVP [23].

The presence of pre-pregnancy thyroid disorders emerged as a statistically significant risk factor, while chronic hypertension and diabetes mellitus (types 1 and 2) were not significant. This finding may be attributed to the small sample size, as the prevalence of thyroid disorders is notably higher among women of reproductive age (20–40 years), while chronic hypertension and diabetes are less prevalent in this demographic [24].

Nausea and Vomiting in Pregnancy also appear to impact pregnancy outcomes. Consistent with the existing literature [1], the NVP group was associated with a higher risk of gestational hypertension. Proper placentation is critical for ensuring adequate maternal–fetal nutrient and oxygen exchange [25]. Impaired placental development can lead to complications such as gestational hypertension, which may be influenced by maternal malnutrition in early pregnancy, limiting trophoblast invasion and vascular remodeling [26]. Inadequate placental perfusion could contribute to hypertensive disorders, potentially explaining the link between hyperemesis and gestational hypertension [27]. Of note, in our sample, all patients who developed gestational hypertension belonged to the mild NVP group, suggesting that even less severe forms of NVP may carry a measurable risk and warrant clinical attention. Thus, a diagnosis of NVP may necessitate closer monitoring to detect early hypertensive disorders, facilitating early high-risk pregnancy identification and potentially reducing maternal–fetal complications and hospitalization costs. To further investigate the clinical significance of symptom duration, we stratified our analysis by early versus continued NVP, defining early NVP as symptoms limited to the first trimester and continued NVP as symptoms persisting into both the first and second trimesters. Although no statistically significant differences emerged between these subgroups, this stratification may offer additional insights into disease progression and risk profiles. Larger sample sizes in future studies may help elucidate whether the duration of symptoms influences obstetric outcomes more markedly.

In our study, NVP did not emerge as a risk factor for gestational diabetes. This finding remained consistent when stratifying GDM cases by treatment status, as no increased risk was observed in either treated or untreated groups. Reduced caloric intake and lower gestational weight gain in women with NVP may enhance insulin sensitivity and reduce the risk of gestational diabetes, although current evidence does not confirm a direct causal relationship between NVP and a reduced incidence of gestational diabetes.

NVP did not act as a risk or protective factor for outcomes such as placental anomalies, hospitalizations for threatened preterm labor, postpartum hemorrhage, labor complications, or fetal growth-related disorders (small-for-gestational-age SGA, fetal growth restriction FGR, or large-for-gestational-age LGA). Similarly, the mode of delivery (spontaneous vaginal delivery, operative delivery, or cesarean section) did not appear to be influenced by NVP, as there was no increase in cesarean section rates in the NVP group compared to the non-NVP group. The cesarean section rate was significantly higher in women without NVP (53% vs. 28%, *p* = 0.03). This may be attributed to our sample, which consisted primarily of women with mild NVP, resulting in normal birth weight neonates and low-risk pregnancies. The same explanation applies to birth complications such as postpartum hemorrhage, which tends to occur more frequently in higher-risk pregnancies.

Regarding fetal and neonatal outcomes, no statistically significant differences were observed between the NVP and non-NVP groups. This may be due to the absence of severe hyperemesis, which is more likely to affect fetal outcomes [28].

According to the literature, we indirectly assessed the impact of medical therapy on quality of life through clinical parameters such as the reduction in symptom frequency and the number of hospital admissions, rather than by using a standardized quality-of-life instrument [29]. The therapy significantly improved the quality of life for women, allowing them to engage in daily activities with fewer limitations compared to those who did not receive treatment [30]. However, no significant differences in maternal, fetal, and neonatal complications were observed between patients who received therapy and those who did not. This may be explained by the fact that the majority of cases in our study were mild, with no instances of severe NVP.

## 5. Strengths and Limitations

The most relevant limitation of this study is the small sample size, which limited the statistical power to detect potentially important differences in outcomes. Moreover, the fact that all participants were recruited from a single clinical center may restrict the generalizability of our findings to broader populations. Another critical limitation lies in the absence of patients who experienced NVP during the third trimester. This gap limits our ability to capture the full temporal profile of the condition and prevents us from evaluating whether symptoms might persist or evolve in the later stages of gestation.

Despite these limitations, the study has several notable strengths. Its prospective design, combined with longitudinal follow-up across different pregnancy stages, allowed for a dynamic assessment of symptom evolution and treatment response. The use of a validated instrument (PUQE) and standardized clinical protocols further ensured consistency and reliability in the evaluation of NVP. This methodology provided valuable insight into the impact of early therapeutic intervention in routine care settings. Looking ahead, future investigations should prioritize multicenter recruitment strategies, encompass a broader spectrum of clinical severity, and extend follow-up beyond delivery to assess possible long-term maternal and neonatal outcomes. Randomized controlled trials will also be essential to determine the most effective treatment strategies for managing moderate-to-severe cases of NVP.

## 6. Conclusions

The study confirms that NVP is a common condition affecting a substantial proportion of pregnant women, with a prevalence of approximately 60% in the study population. The majority of cases were mild, and no severe instances of HG were observed. NVP did not appear to significantly impact neonatal outcomes, birth weight, gestational age at delivery, or mode of delivery. The absence of severe NVP or HG cases may account for the lack of significant differences in adverse maternal, fetal, and neonatal outcomes. Medical treatment enhanced the quality of life for the affected women, enabling them to perform daily activities with fewer limitations. Early pharmacological intervention may contribute to a reduction in hospitalization rates. Importantly, these findings underscore the clinical relevance of early recognition and management of NVP. Even when not associated with adverse obstetric outcomes, these conditions can severely affect maternal well-being and have a considerable impact on the quality of life of both the patient and her family, during pregnancy and even in the postpartum period. If left untreated or managed late, symptoms may worsen, leading to greater physical and psychological burden, and potentially higher healthcare costs due to increased use of emergency services or hospital admissions. Furthermore, the presence of NVP may serve as an early indicator of pregnancies requiring closer clinical monitoring, given their possible association with maternal complications such as gestational hypertension.

## Figures and Tables

**Table 1:** Characteristics of study population.

STUDY POPULATION	TOTAL PATIENT WITH NVP (n = 47)	PATIENTS WITHOUT NVP (n = 32)	*p* Value (Group with vs. Without NVP)	Odds Ratio (IC 95%)
Age (years old)	31.0 ± 3.0	31.0 ± 3.0	0.37	n/a
Body mass index (BMI) pre	21.1 ± 1.8	21.0 ± 2.0	0.42	n/a
Weight increase	11.2 ± 5.8	11.0 ± 5.0	0.74	n/a
Family history	19 40%	5 16%	0.03	3.66 (1.20–11–21)
Menarche (years)	13.2 ± 2.2	13.0 ± 2.0	0.06	n/a
Time since menarche (years)	17.9 ± 3.1	18.0 ± 3.0	0.21	n/a
Gravidity	1.0 ± 0.7	1.2 ± 0.8	0.49	n/a
Parity	0.9 ± 0.8	1.0 ± 1.0	0.02	n/a
Gynecological pathologies	7 15%	4 12%	1.00	1.23 (0.33–4.59)
Thyroid disorders	7 15%	3 9%	0.73	1.69 (0.40–7.10)
Diabetes type 1 and 2	0 0.0%	1 3%	0.41	0.00
Chronic hypertension	1 2%	0 0%	1.00	0.00
Smoke	15 32%	7 22%	0.44	1.67 (0.59–4.73)
Alcohol	11 23%	9 28%	0.79	0.78 (0.28–2.18)
Caucasian ethnicity	9 19%	8 25%	0.58	0.71 (0.24–2.09)
Pma	2 4%	3 9%	0.39	0.43 (0.07–2.73)
Spontaneous delivery	45 96%	29 91%	0.39	2.33 (0.37–14.79)
Single	45 96%	31 97%	1.00	0.73 (0.06–8.36)
Twin	2 4%	1 31%	1.00	1.38 (0.12–15.87)

**Table 2:** Comparative analysis of obstetric complications in pregnant patients with and without nausea and vomiting.

PREGNANCY COMPLICATIONS	PATIENTS WITH NVP (n = 47)	PATIENTS WITHOUT NVP (n = 32)	*p* Value (Group with vs. Without NVP)	Odds Ratio (IC 95%)
Gestational hypertension	7 14%	0 0%	0.04 *	n/a
Preclampsia	2 4%	0 0%	0.51 *	n/a
Eclampsia	0 0%	0 0%	1.00 *	n/a
Cholestasis	1 2%	0 0%	1.00 *	n/a
Gestational hypothyroidism	3 6%	0 0%	0.27 *	n/a
Gestational diabetes	4 8%	9 28%	0.03 *	0.24 (0.07–0.86) *
Placental anomalies	5 11%	2 6%	0.69 *	1.79 (0.32–9.83) *
MPP ricoveri	4 8%	0 0%	0.14 *	n/a
PPH	9 19%	4 12%	0.54 *	1.66 (0.46–5.93) *
Labor abnormalities (I stadio12 h, II > ¾)	6 13%	3/32 9%	0.73 *	1.41 (0.33–6.12) *
Vaginal delivery	28 59%	14 44%	0.18 *	1.89 (0.76–4.70) *
Operative delivery	5 11%	1 3%	0.39 *	3.69 (0.41–33.20) *
Caesarean section	13 28%	17 53%	0.03 *	0.34 (0.13–0.87) *
Induction of labor	10 21%	11 34%	0.21 *	0.52 (0.19–1.42) *
IUGR	5 11%	2 6%	0.69 *	1.79 (0.32–9.83) *
SGA	1 2%	1 3%	1.00 *	0.67 (0.04–11.18) *
LGA	1 2%	2 6%	0.56 *	0.33 (0.03–3.76) *

* Odds ratios, confidence intervals, and *p*-values from univariable logistic regression.

**Table 3:** Comparison of gestational hypertension between early and continued NVP.

Group	Early NVP	Continued NVP	*p* Value (Group Early vs. Continued NVP)	Odds Ratio (IC 95%)
Total (n)	47	17	1.00	0.82 (0.19–3.60)
GHTN cases	7 15%	3 18%		

**Table 4:** Fetal outcomes.

FETAL OUTCOMES	PATIENTS WITH NVP (n = 47)	PATIENTS WITHOUT NVP (n = 32)	*p* Value (Group with vs. Without NVP)	Odds Ratio (IC 95%)
Fetal malformations	5 10%	1 3%	0.39 *	3.69 (0.41–33.20) *
Apgar at birth 1′	9.2 ± 0.9	9.0 ± 1.0	0.60 *	n/a
Apgar at birth 5′	10.0 ± 1.1	10.0 ± 1.0	0.08 *	n/a
Gestational age at birth	39.2 ± 2.1	39.0 ± 2.0	0.45 *	n/a
Weight at birth	3058.0 ± 632.0	3291.0 ± 444.0	0.03 *	n/a
Fetal sex (M)	33 67%	16 48%	0.11 *	2.19 (0.88–5.43) *
Fetal sex (F)	16 33%	17 51%	0.11 *	0.46 (0.18–1.13) *

* Odds ratios, confidence intervals, and *p*-values from univariable logistic regression.

## Data Availability

The original contributions presented in this study are included in the article. Further inquiries can be directed to the corresponding author(s).
